# Comparing Bladder Neck Contracture Rate Between Robotic Intracorporeal and Extracorporeal Neobladder Construction

**DOI:** 10.7759/cureus.56825

**Published:** 2024-03-24

**Authors:** Rohit Bhatt, Dylan J Mittauer, Joel M Vetter, Nimrod S Barashi, Riley McGinnis, Kenneth G Sands, Alexander K Chow, Eric H Kim

**Affiliations:** 1 Department of Urology, University of California Irvine Health, Orange, USA; 2 Department of Urology, Washington University School of Medicine, St. Louis, USA; 3 Department of Urology, Rush University Medical Center, Chicago, USA

**Keywords:** extracorporeal neobladder construction, intracorporeal neobladder construction, urinary diversion, bladder neck contracture, robot-assisted radical cystectomy

## Abstract

Robot-assisted radical cystectomy (RARC) has become more accessible to surgeons worldwide, and descriptions of intracorporeal urinary diversion techniques, such as orthotopic neobladder construction, have increased. In this study, we aim to compare the rate of bladder neck contracture (BNC) formation between RARC and two different urinary diversion techniques. We retrospectively reviewed our institutional database for patients with bladder cancer who underwent RARC with intracorporeal neobladder (ICNB) construction (n = 11) or extracorporeal neobladder (ECNB) construction (n = 11) between 2012 and 2020. BNC was defined by the need for an additional surgical procedure (e.g., dilatation, urethrotomy). Patients who underwent RARC with ICNB (n = 11) were compared to patients who underwent RARC with ECNB (n = 11) across patient characteristics and postoperative BNC formation rates. Kaplan-Meier curves were generated for freedom from BNC based on the neobladder approach and compared with the log-rank test. For patients who received an ECNB, 73% (8/11) developed a BNC; in comparison, none of the patients in the ICNB group experienced a BNC. Kaplan-Meier survival analysis demonstrates the ECNB group’s median probability of freedom from BNC as 1.3 years, while the ICNB group was free of BNC over the study period (p < 0.001). RARC with ICNB creation demonstrated a significantly reduced BNC rate in contrast to RARC with ECNB construction. Longer-term follow-up is needed to assess the durability of this difference in BNC rates.

## Introduction

For patients with invasive or high-risk recurrent non-invasive bladder cancer, radical cystectomy remains the treatment of choice. With advancements in surgical technology and technique, minimally invasive surgeries (MIS) have been established to overcome the challenges associated with traditional open surgery [1]. First described by Menon et al. in 2003, robot-assisted radical cystectomy (RARC) has emerged as a popular minimally invasive alternative to open radical cystectomy (ORC) by way of comparable perioperative, functional, and oncologic outcomes [2-8]. Despite the expanding experience in robotics, most surgeons still opt to perform the urinary diversion component of an RARC extracorporeally due to the technical difficulties associated with bowel reconstruction and operative efficiency when done intracorporeally, especially for neobladder construction [9-11].

As accessibility in robotic surgery continues to grow and techniques continue to evolve, descriptions of intracorporeal urinary diversions, such as the orthotopic neobladder, ileal conduit, and continent cutaneous diversions, have also increased [11-14]. Performing the procedure intracorporeally may have potential benefits in decreasing bowel manipulation, reducing insensible fluid loss, and smaller incisions. However, there is limited data exploring the perioperative and oncologic outcomes between intracorporeal and extracorporeal orthotopic neobladder formation, including the rate of bladder neck contracture (BNC) formation. Therefore, this study aims to evaluate and compare the rate of BNC formation between RARC with intracorporeal neobladder (ICNB) vs. extracorporeal neobladder (ECNB) construction. Secondarily, we compare several important operative, perioperative, and postoperative metrics relating to non-BNC complications and patient recovery.

## Materials and methods

Patients and methods

Upon approval of the Institutional Review Board (#201304085), we retrospectively reviewed our institutional database for patients with bladder cancer who underwent RARC with ICNB construction (n = 11) or ECNB construction (n = 11) between 2012 and 2020. Our analysis includes all such cases during the study period; none were excluded. One surgeon performed all the extracorporeal cases between 2012 and 2016, and one surgeon performed all the intracorporeal cases between 2017 and 2020. Both surgeons involved are fellowship-trained in minimally invasive urologic oncology with extensive experience in robotics and laparoscopy. Prior to the study period, both surgeons had surpassed the learning curve for robotic cystectomy and intracorporeal diversions, having each completed 30 or more cases of this type.

Data collection and analysis

Our study cohort was defined by completion of a urinary diversion without open conversion (intracorporeal) or a urinary diversion requiring open conversion (extracorporeal) after completion of RARC. BNC was defined by a patient-reported reduction in urine flow and/or delayed onset of urination with cystoscopic evidence of fibrotic tissue at the urethral anastomosis requiring an additional surgical procedure (e.g., dilation, urethrotomy). Patient characteristics in both groups were compared including age, body mass index (BMI), Charlson comorbidity index (CCI), history of diabetes, use of neo-adjuvant chemotherapy or Bacillus Calmette-Guerin (BCG) immunotherapy, preoperative staging and grade, operative time, estimated blood loss (EBL), intraoperative blood transfusions, length of hospital stay, postoperative complications, histopathology, final cancer staging, and postoperative BNC formation rates.

To minimize the influence of extraneous factors on the BNC formation rate, all study participants were managed in a standardized fashion postoperatively. Foley catheters were kept in place for four weeks after the procedure and were only removed after a retrograde cystogram demonstrated no urine leakage. Patients were then taught to clean intermittent catheterization (CIC) after every void for the next two weeks. If catheterization volumes were acceptable (i.e., residual volumes < 50 mL), catheterization frequency was decreased each following week with a goal frequency of once every three days. It is noteworthy that all neobladder candidates were counseled on the need for CIC preoperatively. Patients who were unwilling to do so were not considered candidates for a neobladder.

The Wilcoxon rank-sum test was performed to analyze univariate associations of quantitative variables. Univariate associations of qualitative variables were tested using Fisher’s exact test and chi-square test. Kaplan-Meier curves were generated for BNC formation based on the neobladder approach and compared using the log-rank test. All analysis was performed using R version 3.5.2 (The R Foundation for Statistical Computing, Vienna, Austria) [15]. Statistical significance was set at p < 0.05.

Robotic cystectomy technique

For both ECNB and ICNB techniques, we performed an RARC using the da Vinci® robotic surgical system (Intuitive Surgical Inc., Sunnyvale, CA) multiport platform (e.g., Si or Xi) with a 12-mm AirSeal® (ConMed, Largo, FL) assistant port as described previously [16]. Patients received standard postoperative care, with multimodal pain management and advancement to regular diet on day two or as tolerated. The postoperative management of catheters and stents is described above.

Intracorporeal neobladder technique

The surgical technique for RARC with ICNB has been previously described [17]. Briefly, we isolated a 50 cm length of ileum at least 20 cm from the terminal ileum using the Endo-GIA™ stapler (Medtronic, Minneapolis, MN). Next, we put the small intestine back into continuity in a side-to-side functional end-to-end fashion using three loads of the Endo-GIA™. To create the neobladder, we formed a modified Hautmann pouch with 5-cm afferent limbs using a combination of 2-0 Vicryl and 2-0 V-Loc™ (Medtronic) suture in a running fashion. The uretero-neobladder anastomoses were formed using a 4-0 Monocryl suture in a running fashion. Before completing the anastomoses, we placed bilateral single-J ureteral stents, which were externalized to the lower abdomen at the end of the case. The urethro-neobladder anastomosis was performed using a double-armed 3-0 V-Loc™ suture in a running fashion. The integrity of anastomoses was verified with an intraoperative leak test using approximately 150 mL of normal saline. We placed a 22 Fr three-way "hematuria" catheter prior to the completion of the neobladder closure (Table 1).

**Table 1:** Comparison of extracorporeal and intracorporeal techniques

Variable	Extracorporeal	Intracorporeal
Bowel stapler (isolation)	Two loads of GIA™	Two loads of Endo GIA™
Bowel stapler (rejoining)	GIA™, TA™	Three loads of Endo GIA™
Ureteral anastomosis	4-0 Monocryl (interrupted ×6)	4-0 Monocryl (running)
Urethral anastomosis	3-0 Monocryl (double-armed UR-6, SH-1, interrupted ×6)	3-0 V-Loc™ (double-armed, running)
Postoperative ureteral stents	Single-J (externalized)	Single-J (externalized)
Postoperative urethral catheter	Foley (18 Fr)	Lubricath™ (three-way, 22 Fr)
Postoperative suprapubic catheter	18 Fr	None

Extracorporeal neobladder technique

We performed RARC with ECNB using a modified form of the technique described previously [18]. After undocking the robot, an 8-cm lower midline incision is made. We isolated a 50-cm length of small bowel as in the intracorporeal technique described previously. We restored bowel continuity in a side-to-side functional end-to-end fashion using one load of the GIA™ (Medtronic) and one load of the TA™ (Medtronic) stapler. We then formed a Hautmann pouch using 3-0 Vicryl in a running fashion. For the uretero-neobladder anastomoses, we used 4-0 Monocryl in an interrupted fashion with approximately six stitches per ureter. We placed bilateral single-J ureteral stents, which were externalized to the lower abdomen at the end of the case. The urethro-neobladder anastomosis was formed using a double-armed 3-0 Monocryl suture with an SH-1 needle (neobladder side) and UR-6 needle (urethra side) in an interrupted fashion with five total stitches. We then “parachuted” the neobladder down to the urethra over an 18 Fr Foley catheter using a similar approach to the vesicourethral anastomosis in a retropubic prostatectomy [19]. A leak test was performed as described above. Additionally, we placed an 18 Fr suprapubic catheter from the neobladder to the lower abdomen (Table 1).

## Results

Among 22 patients included in the study, 18 (82%) were male and four (18%) were female. The mean age in the ECNB group was 55.6 years and 65.3 years in the ICNB group (p < 0.01). BMI was higher on average in the ECNB group compared to the ICNB group (34.1 vs. 24.5, p = 0.03). Between both groups, CCI scores and rates of diabetes mellitus were similar. There was no significant difference between groups in the proportion of patients who had undergone BCG immunotherapy prior to RARC with urinary diversion. Preoperative staging was higher in the ECNB group in comparison to the ICNB group (p < 0.01). Baseline patient characteristics are located in Table 2.

**Table 2:** Baseline patient characteristics

Characteristic	Extracorporeal (n = 11)	Intracorporeal (n = 11)	p-value
Age (years), mean ± SD	55.6 ± 8	65.3 ± 6.8	0.009
Body mass index (kg/m^2^), mean ± SD	34.1 ±13.7	24.5 ± 4.8	0.034
History of diabetes mellitus (%)	3 (27.3)	6 (54.5)	0.387
Charlson comorbidity index (%)	-	-	0.237
0	5 (45.5)	2 (18.2)	-
1	3 (27.3)	2 (18.2)	-
2	3 (27.3)	7 (63.6)	-
BCG immunotherapy (%)	3 (27.3)	5 (45.5)	0.659
Preoperative staging (%)	-	-	0.007
T0	0	3 (27.3)	-
T1	5 (45.5)	0	-
T2	5 (45.5)	8 (72.7)	-
T3	1 (9)	0	-
Preoperative grade (%)	-	-	0.476
Unknown	0	2 (18.2)	-
High	11 (100)	9 (81.8)	-

Despite similar operative times, the EBL was significantly higher in the ECNB group compared to the ICNB group (872.7 vs. 190.9 mL, p < 0.01), requiring four patients (36%) in the ECNB group to receive intraoperative blood transfusions. Overall, hospital courses, including length and postoperative complications, were comparable between both groups. There were no significant differences in final staging between both groups. Perioperative and functional outcomes are summarized in Table 3.

**Table 3:** Perioperative and functional outcomes

Characteristic	Extracorporeal (n = 11)	Intracorporeal (n = 11)	p-value
Operative time (minutes), mean ± SD	503.4 ± 70.8	512.9 ± 57.3	0.898
Estimated blood loss (mL), mean ± SD	872.7 ± 629.8	190.9 ± 80.1	<0.001
Intraoperative blood transfusion (%)	4 (36.4)	0	0.090
Hospital days, mean ± SD	7.3 ± 2.1	8 ± 2.9	0.641
Postoperative complications (Clavien I-V) (%)	5 (45.5)	2 (18.2)	0.361
Final staging (%)	-	-	0.127
T0	0	4 (36.4)	-
CIS	2 (18.2)	0	-
T1	3 (27.3)	1 (9.1)	-
T2	4 (36.4)	5 (45.5)	-
T3 or T4	2 (18.2)	1 (9.1)	-

**Figure 1:**
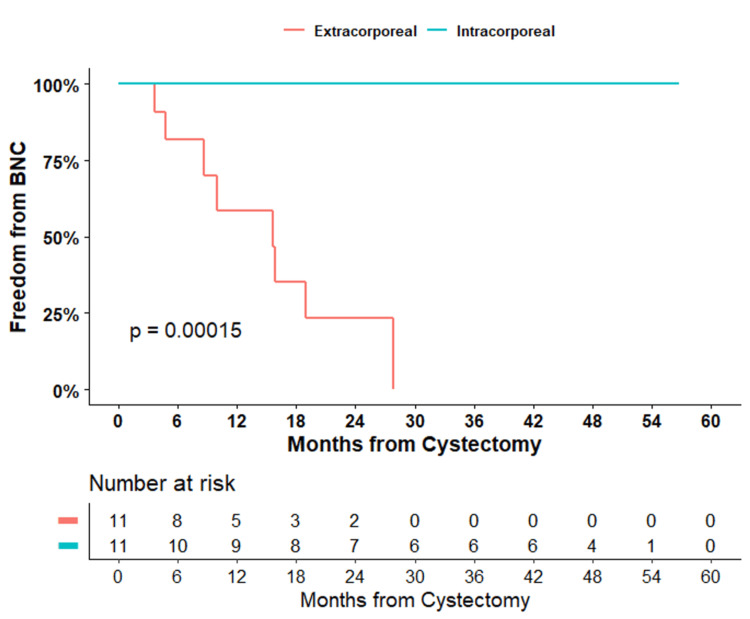
Kaplan-Meier survival analysis for BNC based on the neobladder approach The extracorporeal cohort demonstrates a median time to BNC of 1.3 years compared to the intracorporeal cohort, which is free of BNC.

Based on the Kaplan-Meier survival analysis summarized in Figure 1, patients in the ECNB cohort demonstrated a median time to BNC of 1.3 years over a median follow-up of 31 months, while patients who had undergone an ICNB diversion were free of BNC over a median follow-up of 46 months (p < 0.01).

## Discussion

Despite the increased adoption of RARC and subsequent advancements in the development of intracorporeal techniques, the utility of the intracorporeal approach has been limited primarily due to a steep learning curve and longer operative times [20]. This proportionally modest use at high volume academic centers has therefore led to little published data describing its impact on short and long-term functional outcomes. For this reason, we describe the first comparative study to our knowledge evaluating the rate of BNC formation between patients with extracorporeally and intracorporeally constructed neobladders.

In an era of increased adoption of RARC with orthotopic neobladder construction, an infrequent complication is the formation of a BNC at the junction of the urethra and neobladder, often presenting with increased risk of urinary retention, incontinence, and urinary tract infections. Prior to robotic surgery, BNC formation rates after ORC with ECNB construction and open retropubic prostatectomy (ORP) were reported at 3-9% [21-22] and 2.6-6% [23-24], respectively. Although BNC etiology is unclear, the construction of a water-tight, stress-free anastomosis of the neobladder and urethral mucosal surfaces under direct visualization is key in minimizing the risk of urine leaks and hematomas that may promote scar tissue, a feature that is seen in ORC and ORP approaches [25]. In contrast, the mini-laparotomy required in those undergoing RARC with ECNB diversion is hypothesized to abate many potential perioperative benefits associated with MIS. Due to suboptimal visualization of the urethra through the mini-laparotomy incision in addition to increased bleeding, it is postulated that achieving excellent mucosal apposition becomes more difficult and meticulous, hence increasing the risk of BNC formation [26]. In the present review, eight patients (73%) in the ECNB cohort reflected this risk after developing a BNC over a follow-up of 31 months in comparison to all 11 patients who underwent ICNB diversions and were free of BNC over a 46-month follow-up period. Superior stereoscopic visualization of the urethra, increased degrees of freedom with robotic wrist instruments, and movement refinement controls offered by the intracorporeal technique provided more precise dissection and reduced postoperative systemic inflammation through limited peritoneal cavity exposure to air [26-28]. This alleviates many maneuvering limitations associated with the extracorporeal approach and enables efficient suturing of the mucosal surfaces [11]. Uninterrupted pneumoperitoneum also offers mechanical compression of smaller blood vessels that further diminishes blood loss, which can be seen in the ICNB group’s reduced EBL in comparison to the ECNB group (190.9 vs. 872.7 mL, p < 0.01).

In response to the scarcity of data comparing intracorporeal and extracorporeal urinary diversion techniques, a recent multi-institutional retrospective study of 411 patients by Dalimov et al. comparing the perioperative and oncologic outcomes between these two methods reported similar operative times (p = 0.56), less intraoperative blood loss, overall shorter hospital stays (p < 0.01) with fewer re-operations (p = 0.02), and comparable overall survival (p = 0.21). However, ICNB patients experienced a significantly higher overall hospital readmission rate, with the majority occurring within 30 days of RARC (p = 0.02). Conversely, multivariate analysis demonstrated that high neobladder volume at an institution was associated with reduced odds of readmission, suggesting that, on average, the higher readmission rate could be a byproduct of some surgeons being on the earlier segment of the learning curve [29].

With greater than 30% of the global population estimated to be older than 65 by the year 2030, urologists are and will continue to encounter a larger population of elderly patients requiring bladder cancer treatment. Moreover, this aging population will bring an increased number and complexity of comorbid conditions, which will serve as additional barriers to surgical intervention [30]. Presently, the majority of RARC with neobladder formation surgeries are performed at high-volume academic centers, especially those using intracorporeal techniques. This accumulation of intracorporeal experience amongst robotic teams and surgeons has resulted in the broadening of patient selection criteria to include those that were previously considered sub-optimal surgical candidates (e.g., elderly, multiple comorbidities), a feature which may explain why the ICNB group was on average older than the ECNB group (65.3 vs. 55.6, p < 0.01). Despite the increasing sophistication and volume of neobladder creation at large academic centers, these techniques will need to become available more widely in the community to meet patient demand in the future. Conversely, patients in the ECNB group were associated with a higher BMI and preoperative stage, which may be attributable to each respective surgeon’s patient selection criteria or indicative of the anticipated difficulty of ICNB in patients with larger body habitus. Notably, there was no significant difference in final pathological staging postoperatively.

Our study has several limitations, one being the relatively small sample size for both ECNB and ICNB groups in our single-center cohort. It is important to note that both neobladder reconstructive techniques were relatively novel at the time our two surgeons were performing them. Furthermore, candidacy for a reconstructive procedure of this difficulty requires careful and serious considerations, which act as added filters and further restrict the volume of patients. It is also important to note that BNC is ultimately a clinical diagnosis based on subjective measurements, such as reported symptoms and cystoscopic findings, which could theoretically bias results. Other limitations include potential operator bias and unmeasured confounding variables owing to the retrospective design of the study. The notable strengths of the study include the advanced expertise of both surgeons, robust data collection protocol, and focused comparison between groups.

## Conclusions

Here, we present the results of a single-center retrospective cohort comparing the rate of BNC formation after RARC between two orthotopic neobladder formation techniques. Intracorporeal neobladder formation was associated with a significant reduction in BNC formation rate and intraoperative EBL compared to extracorporeal formation over mean follow-up periods of 46 and 31 months, respectively. Although larger studies over a longer follow-up period are warranted, our findings indicate that intracorporeal continent diversion should be considered over extracorporeal approaches in appropriately selected patients. Future research could, for instance, involve a larger multi-center cohort and directly compare oncologic outcomes between intracorporeal and extracorporeal approaches.

## References

[1] Haber GP, Crouzet S, Gill IS (2008). Laparoscopic and robotic assisted radical cystectomy for bladder cancer: a critical analysis. Eur Urol.

[2] Alfred Witjes J, Lebret T, Compérat EM (2017). Updated 2016 EAU guidelines on muscle-invasive and metastatic bladder cancer. Eur Urol.

[3] Babjuk M, Böhle A, Burger M (2017). EAU guidelines on non-muscle-invasive urothelial carcinoma of the bladder: update 2016. Eur Urol.

[4] Martini A, Touzani A, Ploussard G (2023). Lower detrusor apron-sparing robot-assisted radical cystectomy and intracorporeal neobladder reconstruction: technique and preliminary outcomes. Eur Urol Focus.

[5] Sung HH, Ahn JS, Seo SI, Jeon SS, Choi HY, Lee HM, Jeong BC (2012). A comparison of early complications between open and robot-assisted radical cystectomy. J Endourol.

[6] Bak DJ, Lee YJ, Woo MJ (2016). Complications and oncologic outcomes following robot-assisted radical cystectomy: what is the real benefit?. Investig Clin Urol.

[7] Nix J, Smith A, Kurpad R, Nielsen ME, Wallen EM, Pruthi RS (2010). Prospective randomized controlled trial of robotic versus open radical cystectomy for bladder cancer: perioperative and pathologic results. Eur Urol.

[8] Kader AK, Richards KA, Krane LS, Pettus JA, Smith JJ, Hemal AK (2013). Robot-assisted laparoscopic vs open radical cystectomy: comparison of complications and perioperative oncological outcomes in 200 patients. BJU Int.

[9] Smith AB, Raynor M, Amling CL (2012). Multi-institutional analysis of robotic radical cystectomy for bladder cancer: perioperative outcomes and complications in 227 patients. J Laparoendosc Adv Surg Tech A.

[10] Menon M, Hemal AK, Tewari A, Shrivastava A, Shoma AM, Abol-Ein H, Ghoneim MA (2004). Robot-assisted radical cystectomy and urinary diversion in female patients: technique with preservation of the uterus and vagina. J Am Coll Surg.

[11] Goh AC, Gill IS, Lee DJ (2012). Robotic intracorporeal orthotopic ileal neobladder: replicating open surgical principles. Eur Urol.

[12] Gupta NP, Gill IS, Fergany A, Nabi G (2002). Laparoscopic radical cystectomy with intracorporeal ileal conduit diversion: five cases with a 2-year follow-up. BJU Int.

[13] Chopra S, de Castro Abreu AL, Berger AK, Sehgal S, Gill I, Aron M, Desai MM (2017). Evolution of robot-assisted orthotopic ileal neobladder formation: a step-by-step update to the University of Southern California (USC) technique. BJU Int.

[14] Goh AC, Aghazadeh MA, Krasnow RE, Pastuszak AW, Stewart JN, Miles BJ (2015). Robotic intracorporeal continent cutaneous urinary diversion: primary description. J Endourol.

[15] (2021). R: a language and environment for statistical computing. https://www.R-project.org/.

[16] Hussein AA, Li Q, Guru KA (2022). Robot-assisted radical cystectomy: surgical technique, perioperative and oncologic outcomes. Curr Opin Urol.

[17] Elsayed AS, Aldhaam NA, Nitsche L (2020). Robot-assisted radical cystectomy: review of surgical technique, and perioperative, oncological and functional outcomes. Int J Urol.

[18] Murphy DG, Challacombe BJ, Elhage O, O'Brien TS, Rimington P, Khan MS, Dasgupta P (2008). Robotic-assisted laparoscopic radical cystectomy with extracorporeal urinary diversion: initial experience. Eur Urol.

[19] Reiner WG, Walsh PC (1979). An anatomical approach to the surgical management of the dorsal vein and Santorini's plexus during radical retropubic surgery. Journal Urol.

[20] Giri S, Sarkar DK (2012). Current status of robotic surgery. Indian J Surg.

[21] Patel SG, Cookson MS, Clark PE, Smith JA Jr, Chang SS (2008). Neovesical-urethral anastomotic stricture after orthotopic urinary diversion: presentation and management. BJU Int.

[22] Yu J, Lee CU, Chung JH (2024). Impact of urinary diversion type on urethral recurrence following radical cystectomy for bladder cancer: propensity score matched and weighted analyses of retrospective cohort. Int J Surg.

[23] Breyer BN, Davis CB, Cowan JE, Kane CJ, Carroll PR (2010). Incidence of bladder neck contracture after robot-assisted laparoscopic and open radical prostatectomy. BJU Int.

[24] Kostakopoulos A, Argiropoulos V, Protogerou V, Tekerlekis P, Melekos M (2004). Vesicourethral anastomotic strictures after radical retropubic prostatectomy: the experience of a single institution. Urol Int.

[25] Simhan J, Ramirez D, Hudak SJ, Morey AF (2014). Bladder neck contracture. Transl Androl Urol.

[26] Tan WS, Khetrapal P, Tan WP, Rodney S, Chau M, Kelly JD (2016). Robotic assisted radical cystectomy with extracorporeal urinary diversion does not show a benefit over open radical cystectomy: a systematic review and meta-analysis of randomised controlled trials. PLoS One.

[27] Haseebuddin M, Benway BM, Cabello JM, Bhayani SB (2010). Robot-assisted partial nephrectomy: evaluation of learning curve for an experienced renal surgeon. J Endourol.

[28] Sammour T, Kahokehr A, Hill A (2010). The humoral response after laparoscopic versus open colorectal surgery: a meta-analysis. J Surg Res.

[29] Dalimov Z, Iqbal U, Jing Z (2022). Intracorporeal versus extracorporeal neobladder after robot-assisted radical cystectomy: results from the international robotic cystectomy consortium. Urology.

[30] Yeung C, Dinh T, Lee J (2014). The health economics of bladder cancer: an updated review of the published literature. Pharmacoeconomics.

